# Multimodal Management of a Descending Aorta Injury with Penetrating Chest Trauma: A Case Report

**DOI:** 10.3390/reports7030063

**Published:** 2024-08-01

**Authors:** Giuseppe Sena, Paolo Perri, Paolo Piro, Francesco Zinno, Daniela Mazzuca, Davide Costa, Raffaele Serra

**Affiliations:** 1 Department of Vascular Surgery, A.O.U. “R. Dulbecco”, 88100 Catanzaro, Italy; gspp.sena@gmail.com; 2 Department of Vascular Surgery, “Annunziata” Hospital, 87100 Cosenza, Italy; p.piro@aocs.it; 3 Immunohaematology Section, “Annunziata” Hospital, 87100 Cosenza, Italy; f.zinno@aocs.it; 4 Department of Clinical and Experimental Medicine, University Magna Graecia of Catanzaro, 88100 Catanzaro, Italy; daniela.mazzuca@unicz.it; 5 Interuniversity Center of Phlebolymphology (CIFL), University Magna Graecia of Catanzaro, 88100 Catanzaro, Italy; davide.costa@unicz.it; 6 Department of Medical and Surgical Sciences, University Magna Graecia of Catanzaro, 88100 Catanzaro, Italy

**Keywords:** TEVAR, penetrating chest trauma, descending aorta injury, thoracotomy, mediastinal hematoma, trauma surgery, blood transfusions, emergency transfusions, gunshot-wound-related emergency

## Abstract

A penetrating thoracic aorta injury (PTAI) is a life-threatening condition with significant morbidity and mortality, often resulting from several traumatic mechanisms. Among these, gunshot wounds leading to aortic injury are exceedingly rare and pose unique challenges in terms of diagnosis, management, and surgical intervention. We present a case of a 47-year-old male victim of a gunshot wound resulting in penetrating chest trauma and a descending thoracic aorta injury. This report outlines the sequential management involving thoracic endovascular aortic repair (TEVAR), followed by surgical intervention for hematoma drainage and foreign body removal, highlighting the interdisciplinary approach required in managing complex cardiothoracic injuries.

## 1. Introduction

Penetrating chest trauma can lead to life-threatening injuries, including major vascular trauma. A penetrating thoracic aorta injury (PTAI) is a life-threatening condition with significant morbidity and mortality, often resulting from several traumatic mechanisms. Among these, gunshot wounds leading to aortic injury are exceedingly rare and pose unique challenges in terms of diagnosis, management, and surgical intervention. In the literature, thoracic trauma is usually described either globally or subdivided according to the mechanism of injury or to specific vascular structures, so the general frequency of mediastinal vascular injuries remains unclear. Mortuary-reported incidences are frequently excluded. Mattox reported an 18% incidence in 1989, while Pate reported 93 cases of a penetrating injury in 1993, with a 70% survival rate after repair. A more recent publication found that around 6% of all cases involved blunt vascular injury, with aortic injury being the most common [1,2,3]. Over the past two decades, the assessment and management of patients with mediastinal vascular injuries have advanced substantially. Diagnosis has been improved with multi-detector computer tomographic angiography (CTA), usually protocol based. Additionally, minimally invasive operative strategies and endovascular therapy have become first-line tools in patient care. Immediate diagnosis and intervention are pivotal to patient outcomes. Here, we describe a 47-year-old male with penetrating gunshot trauma of the chest treated with thoracic endovascular aortic repair (TEVAR) as a primary intervention followed by an open thoracotomy. In the management of thoracic aortic injury secondary to penetrating trauma, this methodology remains underexplored.

## 2. Case Presentation 

**Figure 1:**
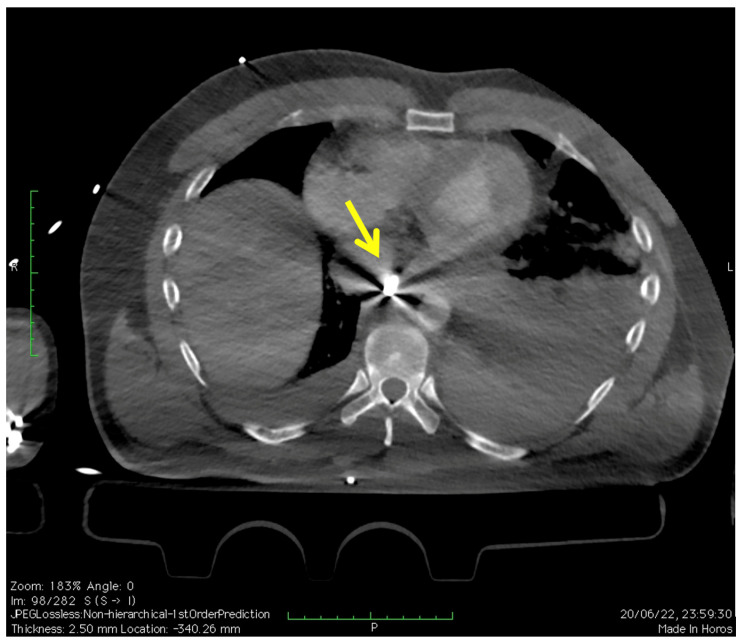
CTA of the chest revealing penetrating thoracic aorta injury (transversal view). Yellow arrow shows the bullet.

A 47-year-old male patient was brought to the emergency room with a left chest gunshot wound. The entry hole was between the eighth and ninth intercostal space posterolateral aspect; the bullet settled in the posterior mediastinum near the esophagus and the descending thoracic aorta. On arrival, the patient was tachypneic, tachycardic, and hypotensive. Immediate resuscitation was initiated, and a focused assessment with sonography for trauma (FAST) was performed, which was inconclusive for free fluid. The chest radiography showed a widened mediastinum and an abnormal left hemithorax opacity, suggesting hemothorax. From the CTA of the chest and the intraoperative angiography it was evident that the aortic lesion and the bullet (yellow arrow) were next to the aorta (Figure 1). 

**Figure 2:**
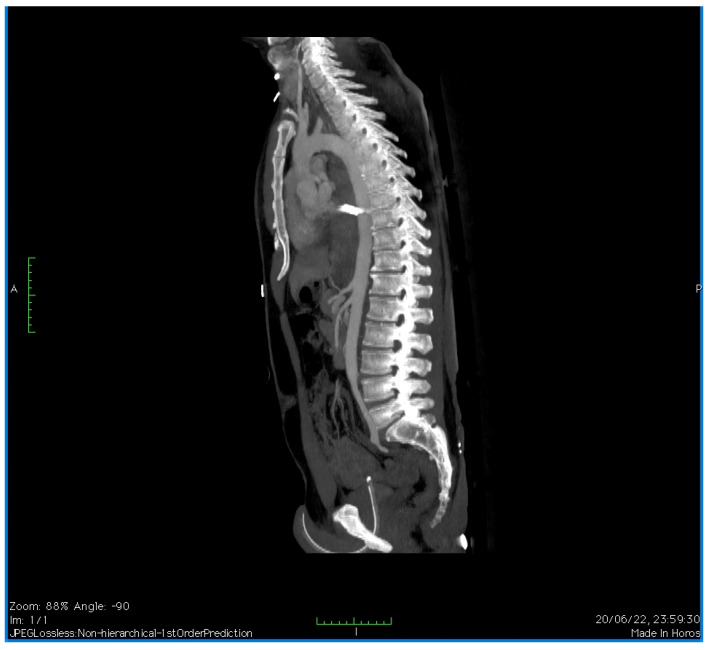
CTA of the chest revealing penetrating thoracic aorta injury (sagittal view).

A CTA of the chest revealed a PTAI with a contained rupture and a large mediastinal hematoma. There was no active extravasation (Figure 2).

**Figure 3:**
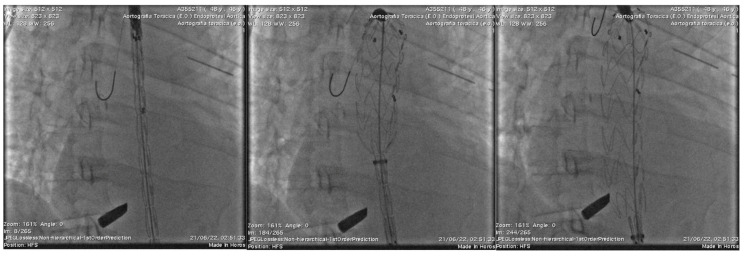
Intraoperative delivery and release phases deployment of Thoracic endovascular stent graft excluding the aortic injury.

The decision was made to proceed with TEVAR as an emergency procedure to stabilize the aortic injury. Under general anesthesia, after puncture of the right femoral artery and catheterization of the aorta, two catheter-directed angiographies (CDAs) were performed, which confirmed the rupture of the descending aorta. A 150 × 24 mm thoracic endovascular stent graft was placed successfully (Valiant Thoracic Captiva VAMF2424C150TE), excluding the aortic injury (Figure 3). Via the right femoral artery access and following the diagnostic angiography with a 5 FR Pigtail catheter, the endoprosthesis was released.

**Figure 4:**
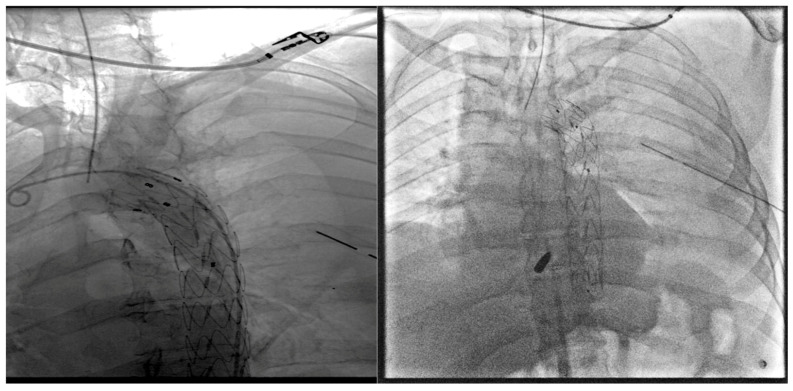
Post-TEVAR angiography showing proper graft placement.

Post-TEVAR angiography confirmed the absence of endoleak and extravasation and the proper graft placement (Figure 4).

**Figure 5:**
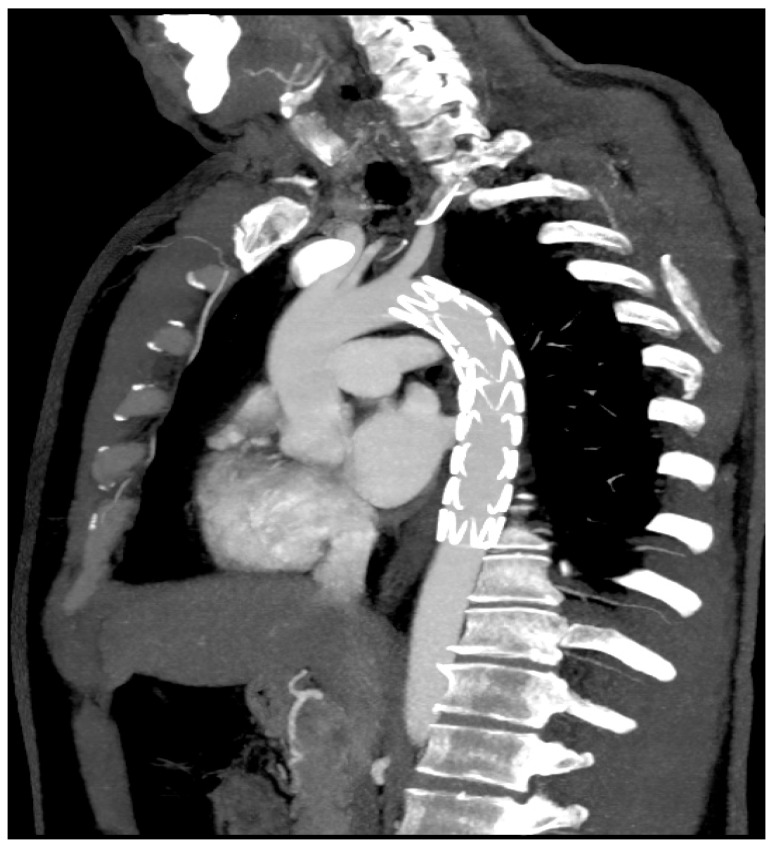
Chest CTA at 1 month after discharge.

Postoperatively, the patient remained hemodynamically stable but developed signs of increasing mediastinal pressure and respiratory distress. A decision was made to perform explorative postero-lateral left thoracotomy in the sixth intercostal space, which revealed a massive hemothorax and a significant mediastinal hematoma after opening the mediastinal pleura. No other anatomical elements were damaged. The presence of a retained bullet lodged near the posterior chest wall, between the descending aorta and the spinal column, was identified. The hematoma and hemothorax were evacuated, and the bullet (caliber 9 mm) was carefully extracted through combined scope and digital maneuvers without further complications. The patient’s recovery was unremarkable, with discharge from intensive care after three days and a return to normal activities ten days following hospital discharge. At home, the patient was administered antibiotic therapy for 14 days: Doxycycline (200 mg/d) and Rifampicin (300 mg/d). Figure 5 shows the chest CTA at 1 month after discharge. 

## 3. Discussion

The first known instance of an aortic injury was recorded by Andreas Vesalius in 1557 [4]. The injury occurred in a victim of a horse-related event and was blunt in nature. Fast forward four centuries later, De Bakey’s group reported successfully repairing an aortic rupture [5]. In the past, a penetrating chest or mediastinum wound often resulted in certain death. Fortunately, current management has improved to the point where open approaches are possible for patients with a possible mediastinal vascular lesion, especially if the patient arrives at the emergency room or trauma room alive. Vascular lesions of the major mediastinal vessels can result from blunt or penetrating trauma, and the management of these lesions depends on the related mechanism and the hemodynamic status of the patient. In cases of a penetrating injury, the entry or exit wounds offer useful information regarding the probable tract location. In contrast, blunt injuries require a higher level of attention, particularly in cases of lesions related to high-energy acceleration–deceleration. In the case of penetrating injuries, it is important to consider the junction of the cervicomediastinal region, as a penetrating injury to zone 1 of the neck can easily damage the intrathoracic vasculature [6]. Additionally, the possibility of an associated aerodigestive lesion should always be taken into account. If bleeding does not stop, immediate surgical intervention is required. In patients with a possible injury, who have “soft” or “hard” symptoms and who have apparently controlled bleeding and seem stable, we should ensure that their hemodynamics are normal and proceed with imaging studies. In the case of actively bleeding patients, initial attempts at digital or Foley catheter control should be attempted [7]. Imaging assessment includes FAST to exclude hemothorax, cardiac tamponade, pneumothorax, or abdominal lesions. FAST is performed in the resuscitation unit, followed by a CTA and, especially if surgical emphysema is detected on the CTA, a swallow study or endoscopy to examine the esophagus, trachea, and main bronchi. [8,9]. Alternatively, a formal CDA may be used. In cases where foreign bodies are retained, they may increase the “scatter” and prevent accurate diagnosis, which is common with retained bullets and knife blades. Intimal flaps determining vascular occlusions, pseudoaneurysms, and arteriovenous fistulae are some of the potential injuries. Active bleeding is caused by uncontrolled laceration and lesions to the arteries and large veins. Trans-axial penetrating lesions are slightly different than those seen with anteroposterior penetrating injuries. Therefore, a detailed assessment of the mechanism of injury, along with proper imaging and evaluation of the aerodigestive tract, is essential to determine the best course of treatment for patients with suspected vascular injuries of the mediastinal great vessels. TEVAR has emerged as the preferred therapeutic option for traumatic aortic injuries (TAI), especially those resulting from blunt trauma, and has replaced open repair over the past twenty-five years. Notably, there are reports of successful treatment of PTAI using this approach [8,9,10]. While several studies have underscored the advantages of TEVAR compared to open repair, there is a dearth of large, randomized control trials to support these findings [11]. Hybrid operating rooms offer several advantages in the management of TAI, allowing surgical teams to prepare for both procedures (endovascular and open). They assure an optimal setting to combine endovascular treatments with open surgical interventions, thereby enabling bridging treatments. Moreover, multi-disciplinary teams can carefully evaluate the patient’s condition and collaborate to provide the best possible care [10]. 

Bleeding is the most common cause of death from perforating gunshot wounds, and it was necessary to urgently resort to the transfusion of 4 units of concentrated leucodepleted red blood cells [12].

There are numerous approaches to emergency transfusion resuscitation, all of which focus on the early use of blood products with allowable hypotension and the minimization of crystalloid infusion. In accordance with the literature and good practices in the event of a hemorrhage, blood transfusions must be started as early as possible [13].

This case illustrates the utility of TEVAR as an effective initial intervention for stabilizing the descending thoracic aorta after penetrating trauma, which potentially obviates the need for immediate, more invasive surgery. However, the subsequent development of complications like compressive hematoma necessitated additional open surgical intervention. The dual approach of TEVAR, followed by targeted thoracotomy for hematoma drainage and foreign body removal, showcases the importance of a flexible, adaptive strategy in trauma care. This hybrid approach allowed for the stabilization of the patient’s cardiovascular status while addressing secondary complications in a controlled fashion. 

## 4. Conclusions

The management of penetrating thoracic injuries with associated aortic involvement requires a multidisciplinary approach and often a combination of endovascular and open-surgical techniques. Immediate TEVAR can be a lifesaving intervention that stabilizes the patient enough to allow for subsequent, more deliberate open procedures. This case adds to the growing literature supporting the integrated utilization of TEVAR in the acute phase of complex thoracic trauma.

## Data Availability

The original data presented in the study are included in the article, further inquiries can be directed to the corresponding authors.
